# Enhanced As(III) and As(V) Adsorption From Aqueous Solution by a Clay Based Hybrid Sorbent

**DOI:** 10.3389/fchem.2019.00913

**Published:** 2020-01-15

**Authors:** Rabelani Mudzielwana, Mugera Wilson Gitari, Patrick Ndungu

**Affiliations:** ^1^Environmental Remediation and Nano Science, Department of Ecology and Resource Management, University of Venda, Thohoyandou, South Africa; ^2^Department of Applied Chemistry, University of Johannesburg, Johannesburg, South Africa

**Keywords:** arsenic removal, clay based hybrid sorbent, adsorption kinetics, adsorption isotherms, thermodynamics

## Abstract

In this study, a hybrid arsenic adsorbent was synthesized through intercalation inorganic and organic surfactant cations onto kaolin clay interlayers. The synthesized adsorbent was characterized X-ray fluorescence (XRF), Fourier Transform Infrared spectroscopy (FTIR), Scanning electron microscopy (SEM), and Brunauer-Emmett-Teller (BET). Batch studies were conducted to determine As(III) and As(V) removal capacity of hybrid sorbent synthesized. It is found that As(III) removal is optimum at pH range of 4-6 while As(V) removal is optimum at pH range 4-8. The data for adsorption kinetics fitted to pseudo second order model implying that adsorption of As(III) and As(V) is chemisorption. The isotherm studies showed a better fit to Langmuir isotherm model indicating that adsorption of both As(III) and As(V) occurred on a mono-layered surface. The maximum adsorption As(III) and As(V) capacity at room temperature as determined by Langmuir model were found to be 7.99 and 7.32 mg/g, respectively. Thermodynamic parameters, ΔG° and ΔH° were found to be negative indicating that adsorption process occurred spontaneously and exothermic. Inorgano-organo modified kaolin clay was successfully regenerated for up 7 adsorption-regeneration cycles using 0.01 M HCl as regenerant. This study concluded that hybrid sorbent synthesized in this study is suitable for arsenic removal from groundwater.

## Introduction

Arsenic is a toxic element that is widely distributed in different matrixes within the natural environment. In groundwater, arsenic is mainly introduced through weathering of arsenic bearing rock minerals such as; arsenopyrite (FeAsS), realgar (AsS), cobaltite (CoAsS), and scorodite (FeAsO_4_.2H_2_O) (Smedley and Kinniburgh, 2002). Other anthropogenic activities such as; mining, burning of fossil fuel and agriculture also result in groundwater contamination by arsenic (Zhang et al., 2017). Arsenic occurs in both organic and inorganic form. The inorganic form of arsenic is dominantly found in water and it exist as arsenite [As(III)) and arsenate (As(V)] depending on oxidation state. The arsenate is found in oxidizing conditions while the arsenite is found under reducing conditions (Duker et al., 2005). Arsenite is highly toxic and mobile compared to arsenate (Qi et al., 2015).

Long term exposure to arsenic through drinking water is linked to arsenicosis disease which is manifested by different types of cancer, hypertension, neurological complications and cardiovascular disease (Bhowmick et al., 2018). More than 200 million cases of these diseases has been reported in countries like India, Bangladesh, Argentina, Taiwan, Mexico, and China where elevated concentration of arsenic has been reported (Bardach et al., 2015; Ghosh et al., 2019). The World Health Organization (WHO) has set the provisional guideline for arsenic in drinking water at 10 μg/L (WHO, 2017). Technologies such as precipitation and coagulation, oxidation, reverse osmosis, ion-exchange, and adsorption are generally used for removal of arsenic to acceptable levels (Ghosh et al., 2019). Amongst these technologies, adsorption is widely preferred due to its cost effectiveness, ease of operation and higher efficiency (Qi et al., 2015). Materials such as activated alumina, activated carbon, graphene oxides, clay soils, bone char, and granular ferric hydroxides have been evaluated for their efficiency toward arsenic removal. Although these materials have shown good potential for use in arsenic removal, not all of them can be regenerated effectively and some operate at a narrow pH level which limit their application.

Clay and their minerals are used in removal of arsenic and other contaminants from water because of their properties such as chemical and mechanical stability, higher cation/anion capacity, and higher surface area (Bhattacharyya and Gupta, 2008). Furthermore, their sorption efficiency may be enhanced through modification by high density inorganic polycations and organic surfactants (Gitari and Mudzielwana, 2018). Mishra and Mahato (2016) reported enhanced arsenic adsorption efficiency of bentonite clay modified using iron and manganese oxides while octadecyl benzyl dimethyl ammonium modified bentonite prepared by Su et al. (2011) also showed better As(III) and As(V) adsorption capacity as compared to bare bentonite. Inorgano-organo modified clay mineral has received greater attention from researchers due to their important features such as, possession of two sorption sites which in turn enhances the sorption capacity and also good settling property (Tiwari and Lee, 2012). However, most of the inorgano-organo modified clay adsorbent have shown better sorption efficiency toward As(V) as compared to As(III) which is highly mobile and toxic. Manganese and iron are closely related elements in terms of their chemical properties and occurs concurrently in nature. Manganese oxides are known for their ability to adsorb and oxidize As(III) to As(V) while Fe oxides have greater potential to adsorb As(V) species (Zhang et al., 2012). Therefore, the present investigation aims at developing a novel clay based hybrid adsorbent for As(III) and As(V) removal by modifying kaolin clay mineral with Fe-Mn oxides and HDTMA-Br cationic surfactant.

## Materials and Methods

### Materials

Natural kaolin clay was collected from Limpopo, South Africa. FeCl_3_, MnCl_2_.4H_2_O, NaOH, AsNaO_2_, and HAsNa_2_O_4_ were purchased from Rochelle Chemicals & Lab Equipment CC, South Africa Ltd. Hexadecyltriammonium bromide (HDTMA-Br) was purchased from Merck chemicals, South Africa. All chemicals were of analytical grade and they were used without further purification. Milli-Q water (18.2 MΩ/cm) produced from Millipore system was used for rinsing and preparation of solutions.

### Synthesis of Clay Based Hybrid Sorbent

Inorgano-organo hybrid clay based sorbent was synthesized as follows: 0.25 M FeCl_3_ and 0.25 M MnCl_2_.4H_2_O were prepared by dissolving a known amounts of FeCl_3_ and MnCl_2_.4H_2_O into 250 mL volumetric flasks. Extracts of the respective solutions were mixed at a volume ratio of 3:1 in 250 mL plastic bottle and 1 g of raw kaolin clay (RK) was added and soaked for 10 min. Thereafter, pH of the solution was adjusted to 8.5 by adding 10 mL of 2 M NaOH drop wise into each of the bottles to precipitate Fe^3+^ and Mn^2+^ into their respective oxides. Thereafter, 100 mL of 5 mM HDTMA-Br was added to the mixture and agitated for 60 min at 250 rpm and then aged for 62 h. After aging, the mixture was centrifuged at 3,000 rpm. Residues were washed with Milli-Q water to remove excess supernatants then oven dried for 12 h at 60°C. The modified clay was then milled to pass through 250 μm sieve and then stored in a zip lock plastic bag. The hybrid sorbent was then designated IOK.

### Characterization

Handheld x-ray fluorescence (XRF, S1 titan 600, Bruker, Berlin, Germany) was used to determine the elemental composition, surface chemistry of the adsorbent was determined using Fourier Transform Infra-red spectrum-attenuation total reflectance (FTIR-ATR) (Bruker, Germany) at wavelength range 450 to 4,500 cm^−1^. The pore size distribution, pore volume and surface area were determined using Barrett Joyner Halenda (BJH) (micrometrics ASAP 2020, Norcross, GA, USA) and Brunauer Emmett Teller (BET) (micrometrics Gemini 2375, Norcross, GA, USA) models, respectively. The morphology was determined using scanning electron microscopy (SEM) (Leo1450 SEM, Voltage 10 kV, working distance 14 mm, Ramsey, NJ, USA).

### Batch Experiments

Stock solutions containing 1,000 mg/L As(III) and As(V) were prepared by dissolving an appropriate amounts of AsNaO_2_ and HAsNa_2_O_4_ in Milli-Q water (18.2 MΩ/cm). Solutions were preserved through the addition of few drops of 3 M HNO_3_. Appropriate dilutions were made from the stock solution to prepare working solutions. The adsorption kinetics were evaluated by varying time from 10 to 120 min. Adsorbent dosage of 0.1 g/100 mL and adsorbate concentration of 0.5 mg/L were used. To evaluate the adsorption isotherms, the initial concentration of As(III)/As(V) was varied from 0.5 to 30 mg/L and the adsorbent dosage of 0.1 g/100 mL and contact time of 60 min were maintained. The experiment was carried out at a temperature of 298, 323, and 343 K. The obtained data was used to evaluate the adsorption thermodynamics. The effect of initial pH was evaluated by varying solution pH from 2 to 12 using 0.01 M NaOH and 0.01 M HCl to adjust the pH. The initial adsorbate concentration of 0.5 mg/L, contact time of 60 min and adsorbent dosage of 0.1 g/100 mL were used. The influence of co-existing ions (${\text{F}}_{,}^{-}$ Cl^−^, ${\text{NO}}_{3}^{-}$, ${\text{CO}}_{3}^{2-}$, ${\text{SO}}_{4}^{2-}$, Mg^2+^, and Ca^2+^) was evaluated by spiking 5 mg/L of each co-existing ions in a solution containing 0.5 mg/L of As(III)/As(V). The adsorbent dosage of 0.1 g/100 mL and 60 min contact time were used. All experiments were conducted in triplicate and the mean values were reported. Unless otherwise stated, experiments were conducted at room temperature and initial pH of 6 ± 0.5.

### Adsorbent Regeneration-Reuse Cycles

To evaluate the regeneration and reuse potential of the adsorbent, As(III)/As(V) removal experiment was conducted by treating solution containing 0.5 mg/L As(III)/As(V) with 1.0 g of IOK at initial pH of 6 for 60 min. After agitation, mixtures were filtered through 0.45 μm filter membranes and the residuals of As(III) and As(V) were analyzed. Residues were washed with Milli-Q water and oven dried for 12 h at 60°C and then regenerated using 100 mL of 0.01 M HCl by agitating the mixture for 60 min. The obtained residues were rinsed with excess of Milli-Q water and oven dried for 12 h at 60°C. Thereafter, they were pulverized with a mortar and pestle to pass through 250 μm sieve. After regeneration, As(III)/As(V) removal experiment was conducted as in other experiments. The regeneration-reuse cycle were continued up to 7th cycle.

### Analysis of Residual Arsenic

The residual As(III)/As(V) concentration was measured using ScTRACE Gold electrode attached to 884 professional VA Polarography (Metrohm, SA). A composite solution containing 1 mol/L sulfamic acid, 0.5 mol/L citric acid and 0.45 mol/L KCl was used an electrolyte. For total As concentration, KMnO_4_ was added as an oxidizing agent.

## Results and Discussion

### Physicochemical Characterization

#### Elemental Composition

Table 1 present a comparison of the elemental composition between the RK and IOK. It is observed that SiO_2_ and Al_2_O_3_ are major oxides the kaolin clay mineral. After modification their contents of reduced from 57.1 and 22.05% to 32.29 and 8.75%, respectively. Conversely, Fe_2_O_3_ and MnO increased from 3.88 and 0.02% to 9.31 and 1.23%, respectively.

**Table 1:** Elemental composition of RK and IOK.

**Oxides**	**RK (%w/w)**	**IOK (%w/w)**
SiO_2_	57.1	32.29
Al_2_O_3_	22.05	8.74
Fe_2_O_3_	3.88	9.31
MgO	0.57	0.74
MnO	0.02	1.23
CaO	0.95	0.21
K_2_O	0.16	0.08
TiO_2_	1.76	0.82
P_2_O_5_	0.02	0.012

### FTIR Analysis

**Figure 1:**
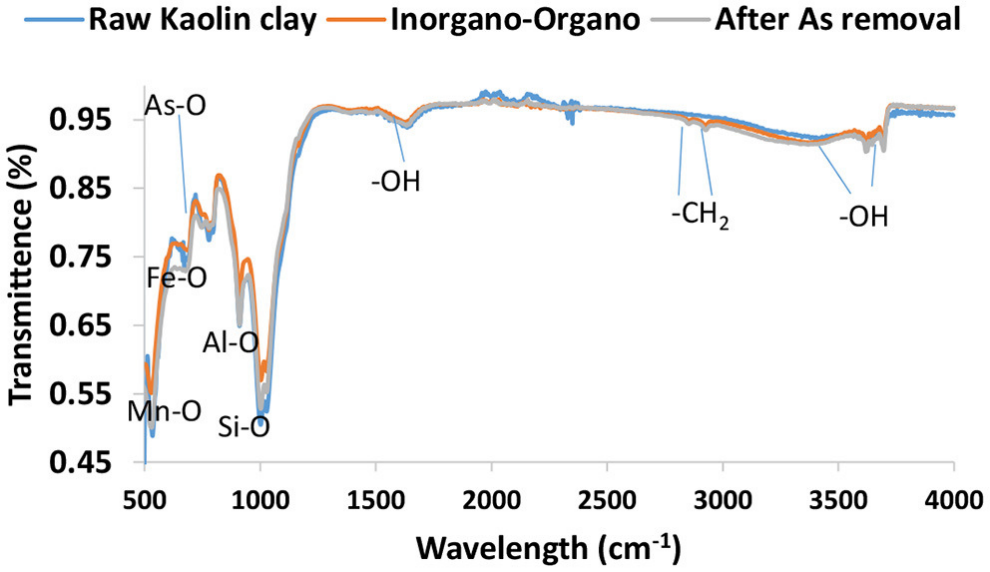
FTIR spectrum of RK, IOK before and after arsenic adsorption.

Figure 1 presents the FTIR spectrum of RK, IOK before and after arsenic removal. The bands at 3,453 and 1,645 cm^−1^ are ascribed to the vibration and stretching of hydroxyl groups and water molecules within the clay interlayers. The prominent IR peaks at wavelength region of 1,030 cm^−1^ could be due to the vibration of Si-O bonds. The bands at 906, 790, and 540 cm^−1^ could be due to the vibration of Al-O, Mn-O, and Fe-O, respectively. After modification by new bands were observed at 2,930 and 2,846 cm^−1^ indicating the presence of –CH_2_ bonds which confirm the introduction of HDTMA-Br within the clay interlayers (Thanhmingliana and Tiwari, 2015). Furthermore, the intensity of bands at 1030, 906, 790, and 540 cm^−1^ increased. This could be attributed to increased concentration of Fe_2_O_3_ and MnO contents as confirmed by XRF analysis. After arsenic removal a new band was observed at 778 cm^−1^ which could be ascribed to As-O bond. The intensity of bands at other wavelength ranges decreased after arsenic removal. This confirms the ion exchange between the hydroxyl groups in the clay interlayers and arsenic species and surface complexation between arsenic and Fe, Mn, Al, and other metals in the surface of the clay minerals.

#### Morphological Analysis

**Figure 2:**
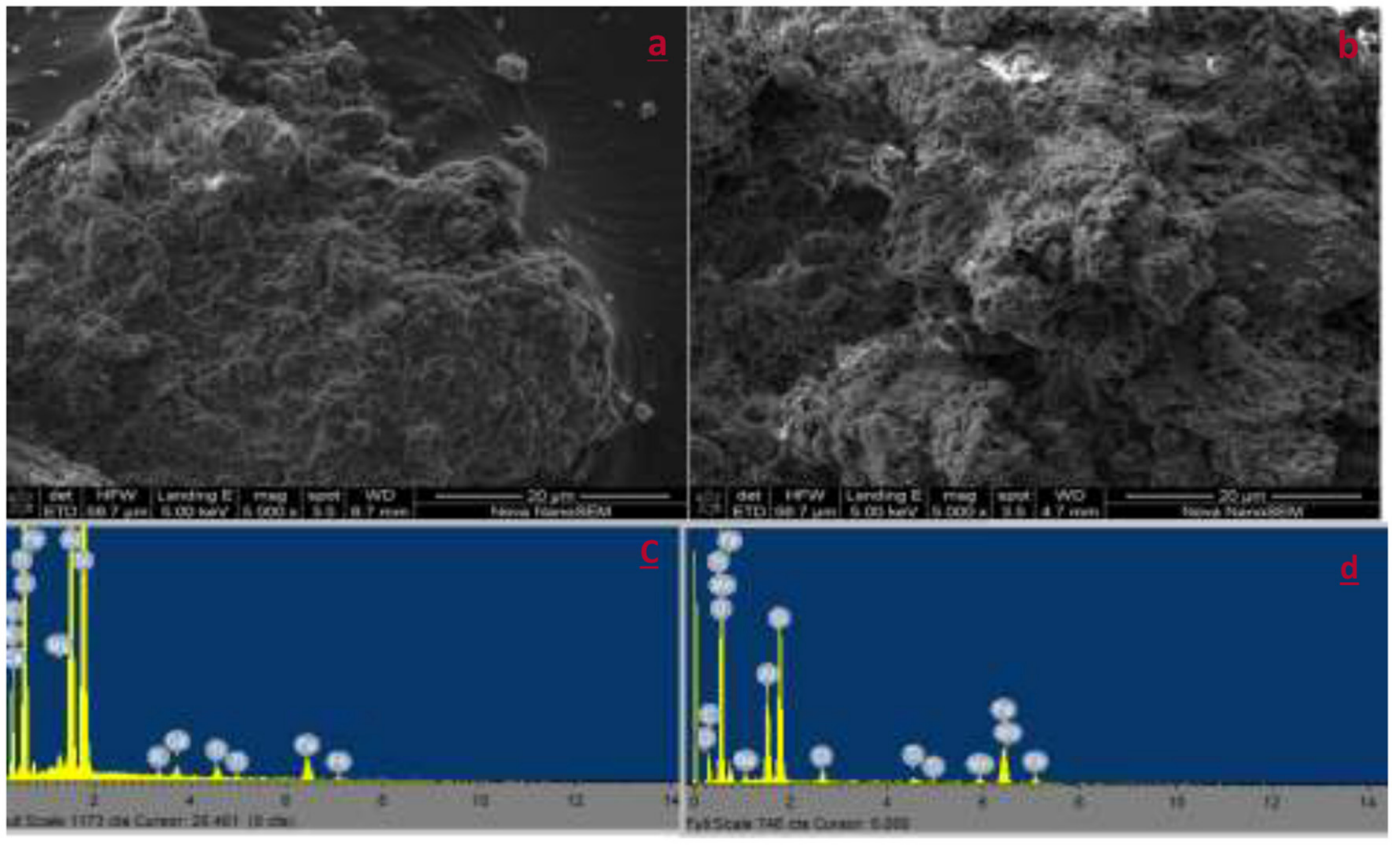
SEM micrographs and SEM-EDS spectrum of RK **(a,c)** and IOK **(b,d)**.

Figure 2 presents the SEM micrographs of RK and IOK. No significant difference observed in the raw and modified kaolin clay mineral. The raw kaolin clay mineral has spongy like rough and porous surface with some irregular shapes. After modification, micrographs shows larger pores. This could be attributed to swelling and expansion of the clay interlayers during modification. The SEM-EDS spectrums of RK shows the presence of Fe, Al, Si, Mg, Ti, K Ca, and C. The spectrum of IOK showed a new peak showing Mn was observed.

### Surface Area Analysis

The surface area and pore analysis are summarized in Table 2. It is noted that the total BET surface area of kaolin clay increased from 19.02 to 87.51 m^2^/g after modification with Fe^3+^ and Mn^2+^ polycations and HDTMA surfactant. Furthermore, the pore volume increased from 0.04 to 0.09 cm^3^/g after modification. The increase in surface area and pore volume could provide more active sites for sorption of ions leading to higher sorption capacity. The average pore size decreased from 9.54 to 4.68 nm after modification. The pore diameter within 2 and 50 nm indicates mesopore nature of the material.

**Table 2:** Surface area and pore analysis.

	**Surface area (m^**2**^/g)**	**Pore volume (cm^**3**^/g)**	**Pore diameter (nm)**
RK	19.02	0.04	9.54
IOK	87.51	0.09	4.68

### Batch Experiments

#### Effect of pH

**Figure 3:**
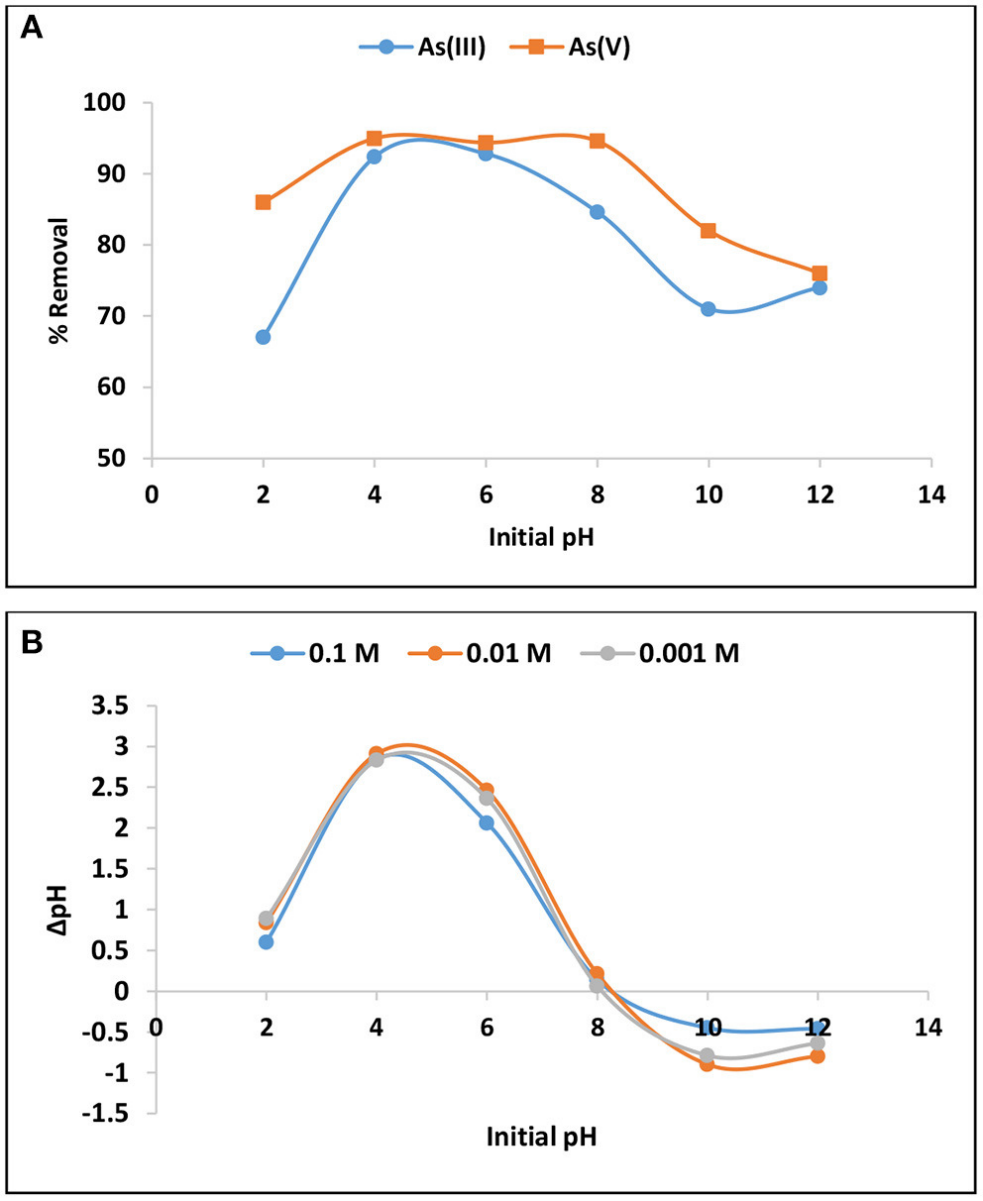
**(A)** Variation of As(III) and As(V) percentage removal with initial pH and **(B)** point of zero charge (pHpzc).

The effect of pH in As(III) and As(V) removal is presented in Figure 3A. The percentage As(V) removal was optimum at pH between 4 and 8 while adsorption of As(III) was optimum at pH range 4 to 6. Arsenic species exist in different forms under various pH levels and its adsorption is influences by the net surface charges. Therefore, to further elucidate the behavior of As(III) and As(V) at various pH levels the pH point of zero charge (pHpzc) of the material was determined using titration method. The results showed that the hybrid material prepared in this study has pHpzc of 8 ± 0.5 (Figure 3B). The material carries net positive charges at pH below pHpzc and net negative charges at pH above pHpzc. Table 3 depicts different speciation of arsenic determined using Visual MINTEQ Version 3.0 at different equilibrium pH levels. Therefore, the decrease in percentage arsenic removal at strong alkaline pH where the surface is negatively charged, could be attributed to electrostatic repulsion since both As(III) and As(V) exist as negatively charged species such as ${\text{HAsO}}_{4}^{2-}$, ${\text{AsO}}_{4}^{3-}$, H_2_${\text{AsO}}_{3}^{-}$, and ${\text{HAsO}}_{3}^{2-}$. The decrease as the pH goes to 2 could be attributed to the fact that these species exist as neutrally charged H_3_AsO_4_ and H_3_AsO_3_ making it difficult to remove via electrostatic attraction to positively charged surface (Lee et al., 2015). Equation 1 to 5 hypothesizes the adsorption of As(III) and As(V).

(1) $$\begin{array}{l} \equiv MOH+H_{3}As_{3}↔\equiv MH_{2}As_{3}+H_{2}O \end{array}$$

(2) $$\begin{array}{l} \equiv MOH+H_{2}As_{3}^{-}↔\equiv MHAs_{3}^{-}+H_{2}O \end{array}$$

(3) $$\begin{array}{l} \equiv MOH+H_{3}AsO_{4}↔\;\equiv MH_{2}AsO_{4}+H_{2}O \end{array}$$

(4) $$\begin{array}{l} \equiv MOH+H_{2}AsO_{4}^{-}↔\equiv MHAsO_{4}^{-}+H_{2}O \end{array}$$

(5) $$\begin{array}{l} \equiv MOH+HAs_{4}^{2-}↔\equiv MAs_{4}^{2-}+H_{2}O \end{array}$$

**Table 3:** Arsenic species at different equilibrium pH levels.

**Eq. pH**	**H_**3**_AsO_**4**_**	**H_**2**_${\text{AsO}}_{4}^{-}$**	**${\text{HAsO}}_{4}^{2-}$**	**${\text{AsO}}_{4}^{3-}$**	**H_**3**_AsO_**3**_**	**H_**2**_${\text{AsO}}_{3}^{-}$**	**${\text{HAsO}}_{3}^{2-}$**
3.62	4.50	95.44	0.042	–	100.00	–	–
6.41	–	78.92	21.07	–	99.82	0.174	–
6.96	–	51.25	48.78	–	99.38	0.61	–
7.02	–	47.78	52.21	–	99.29	0.76	–
9.46	–	0.32	99.17	0.47	33.67	66.31	–
11.53	–	–	59.38	40.61	0.41	99.27	0.30

#### Adsorption Kinetics

**Figure 4:**
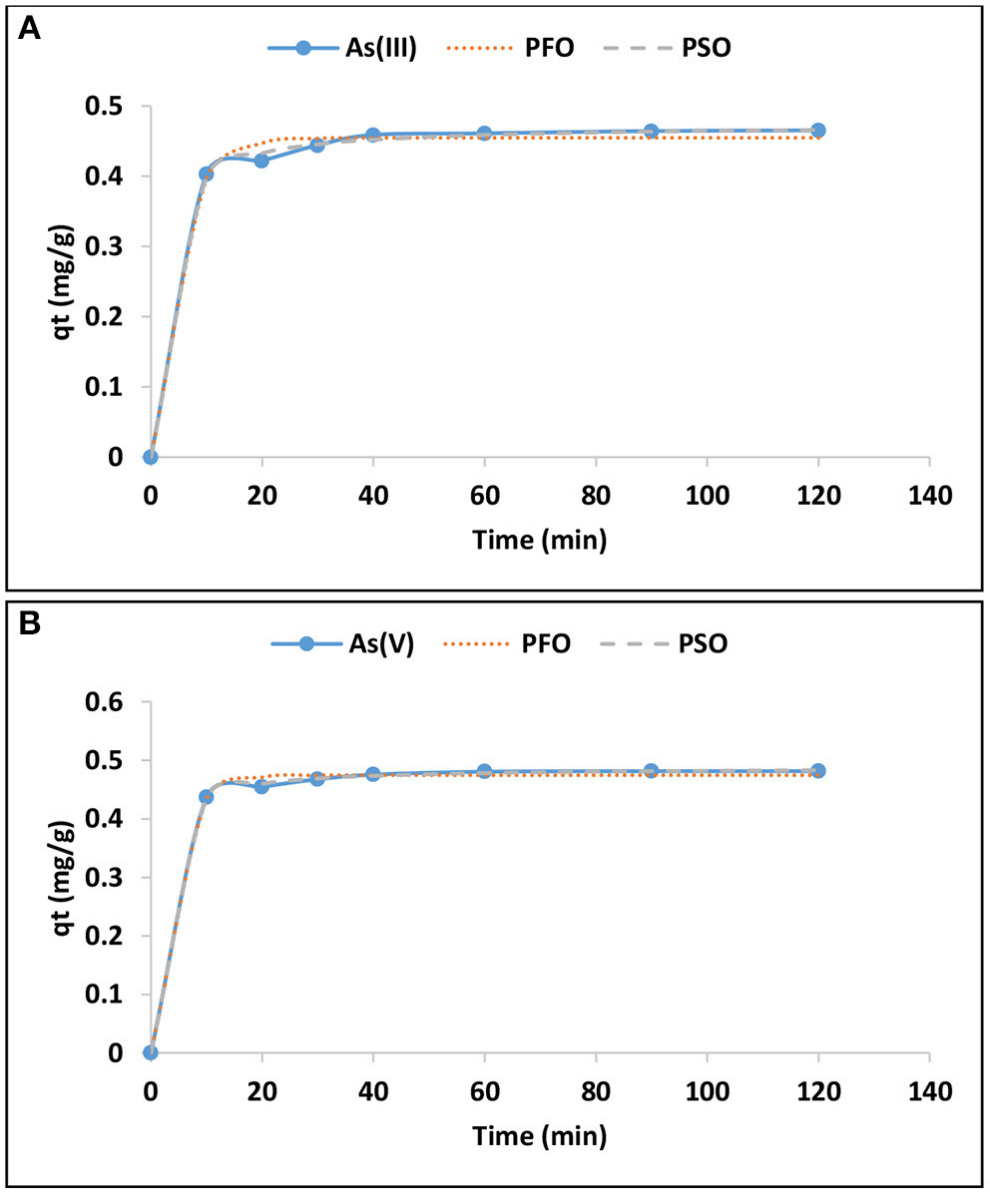
Variation of adsorption capacity of As(III) **(A)** and As(V) **(B)** with time and adsorption kinetics (0.1 g/100 mL adsorption dosage, 0.5 mg/L adsorbate concentration, pH 6.5 ± 0.2).

Adsorption kinetics studies were performed in order to predict the rate of adsorption and to give insight in the rate limiting factor and the adsorption mechanism. Figures 4A,B presents the variation of As(III) and As(V) adsorption capacity with time. The adsorption capacity increased rapidly within the first 40 min and then proceed at slow rate up to 120 min suggesting that the system has reached equilibrium. The same trend was observed for arsenic species.

The pseudo first and second order of reaction kinetics models were used to predict the rate and the mechanism of As(III) and As(V) adsorption onto Inorgano-organo modified kaolin clay mineral. The mathematical representation of the models are depicted in equation 1 and 2, respectively (Qi et al., 2015; Munagapati and Kim, 2017).

(1) $$\begin{array}{l} q_{t}=q_{e}\left(1-e^{{-k}_{1}t}\right) \end{array}$$

(2) $$\begin{array}{l} q_{t}=\frac{q_{e}^{2}k_{2}t}{1+k_{2}q_{e}t} \end{array}$$

Where q_e_ and q_t_ are the adsorption capacities (mg/g) of the sorbent at equilibrium and at any given time, t (min), respectively; K_1_ (min^−1^) and K_2_ (g/mg.min) are the pseudo first order and second order rate constants for adsorption processes, respectively. The initial adsorption rate, h (mg/g.min^−1^) was determined based on the value of qe^2^ (mg/g) and K_2_ (g/mg.min) from the pseudo second order model. The non-linear plots for As(III) and As(V) are presented in Figures 4A,B, respectively, while the constants parameters are presented in Table 4.

**Table 4:** Parameters for pseudo first and second order reactions.

	**Pseudo first order**			**Pseudo second order**			
	**q_**e**_ (mg/g)**	**K_**1**_ (min^**−1**^)**	**R^**2**^**	**q_**e**_ (mg/g)**	**K_**2**_ (g/mg.min)**	**h (mg/g.min^**−1**^)**	**R^**2**^**
As(III)	0.45	0.20	0.70	0.47	1.12	0.26	0.94
As(V)	0.47	0.24	0.80	0.48	1.67	0.38	0.98

The *R*^2^-values for pseudo second order for As(III) and As(V) were found to be 0.94 and 0.98, respectively higher than those pseudo first order (0.70 and 0.80). The values for theoretical adsorption capacity of pseudo second order were found to be higher compared to those from pseudo first order (Table 4). This implies that the adsorption data fitted better to pseudo second order of reaction kinetics. Better fitting to pseudo second order suggests the dominance of chemisorption during the adsorption of As(III) and As(V) onto the hybrid adsorbent.

To further elucidate the rate limiting steps, the adsorption kinetics data was fitted to intra-particle diffusion model of Weber Morris (Weber and Morris, 1963). Equation (3) presents the linearized form of intra-particle diffusion model.

(3) $$\begin{array}{l} q_{t}=k_{i}t^{0.5}+C \end{array}$$

**Figure 5:**
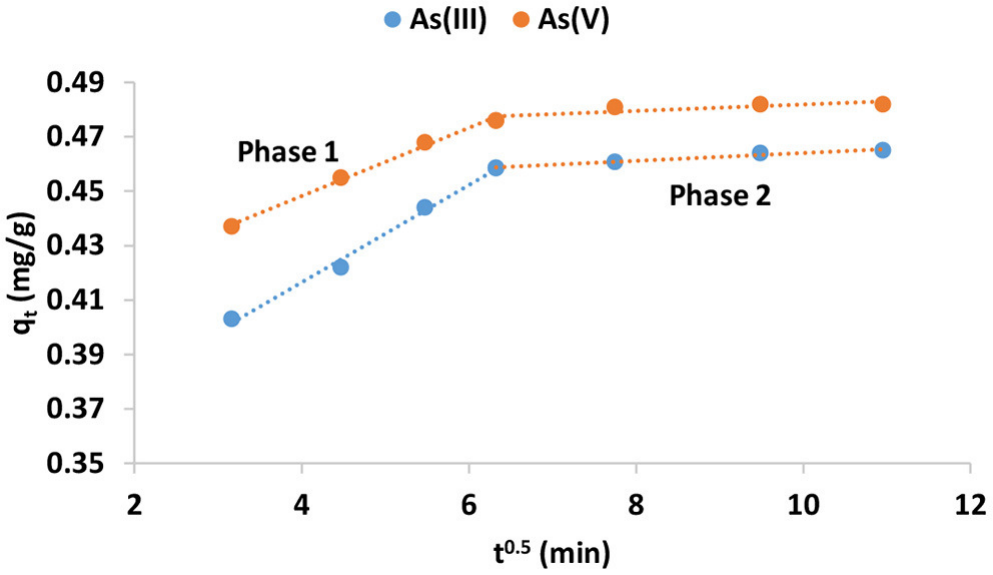
Weber-Morris intra-particle plot for As(III) and As(V) adsorption onto IOK.

Where q_t_ (mg/g) is the adsorption capacity at a given time, t (min); K_i_ is the rate of intra-particle diffusion model C (mg/g) is the constant associated with the thickness of the boundary layer. If the plot of q_t_ against t^0.5^ is linear or passes through origin, the adsorption is solely governed by intra-particle diffusion. However, if the plot yields two or more linear plots, then adsorption is governed by both surface and intra-particle diffusion. The intra-particle plot and the constant parameters for adsorption of As(III) and As(V) is presented in Figure 5 and Table 5, respectively.

**Table 5:** Constant parameters for Weber-Morris intra-particle model.

	**Phase 1**			**Phase 2**		
	**K_**1**_**	**C_**1**_**	**R^**2**^**	**K_**2**_**	**C_**2**_**	**R^**2**^**
As(III)	0.013	0.39	0.99	0.0012	0.47	0.97
As(V)	0.017	0.34	0.97	0.0014	0.44	0.70

The plot (Figure 5) for both species yielded bilinear plots indicating that adsorption of As(III) and As(V) by synthesized adsorbent is a complex process involving both physical and chemical interactions between the adsorbate and the adsorbent. Ryu et al. (2017) observed the same trend during adsorption of As(III) and As(V) onto Fe-Mn modified activated carbon. Phase 1 the film diffusion leading to physiosorption wherein As(III) and As(V) ions are attracted to the boundary layer of the adsorbent through electrostatic attraction forces. Phase 2 reflects the intra-particle diffusion wherein As(III) and As(V) ions diffuse into the pores of the adsorbent leading to chemisorption. This phase involves ion exchange between the hydroxyl ions and arsenic species and weak hydrogen bonding. The rate constant for surface adsorption at phase 1 was found to be higher than the adsorption rate at phase 2 (Table 5). This suggest that intra-particle diffusion is a slower process compared to surface adsorption. This results suggest that adsorption of As(III) and As(V) is a complex process involving both surface and intra-particle diffusion.

#### Adsorption Isotherms

**Figure 6:**
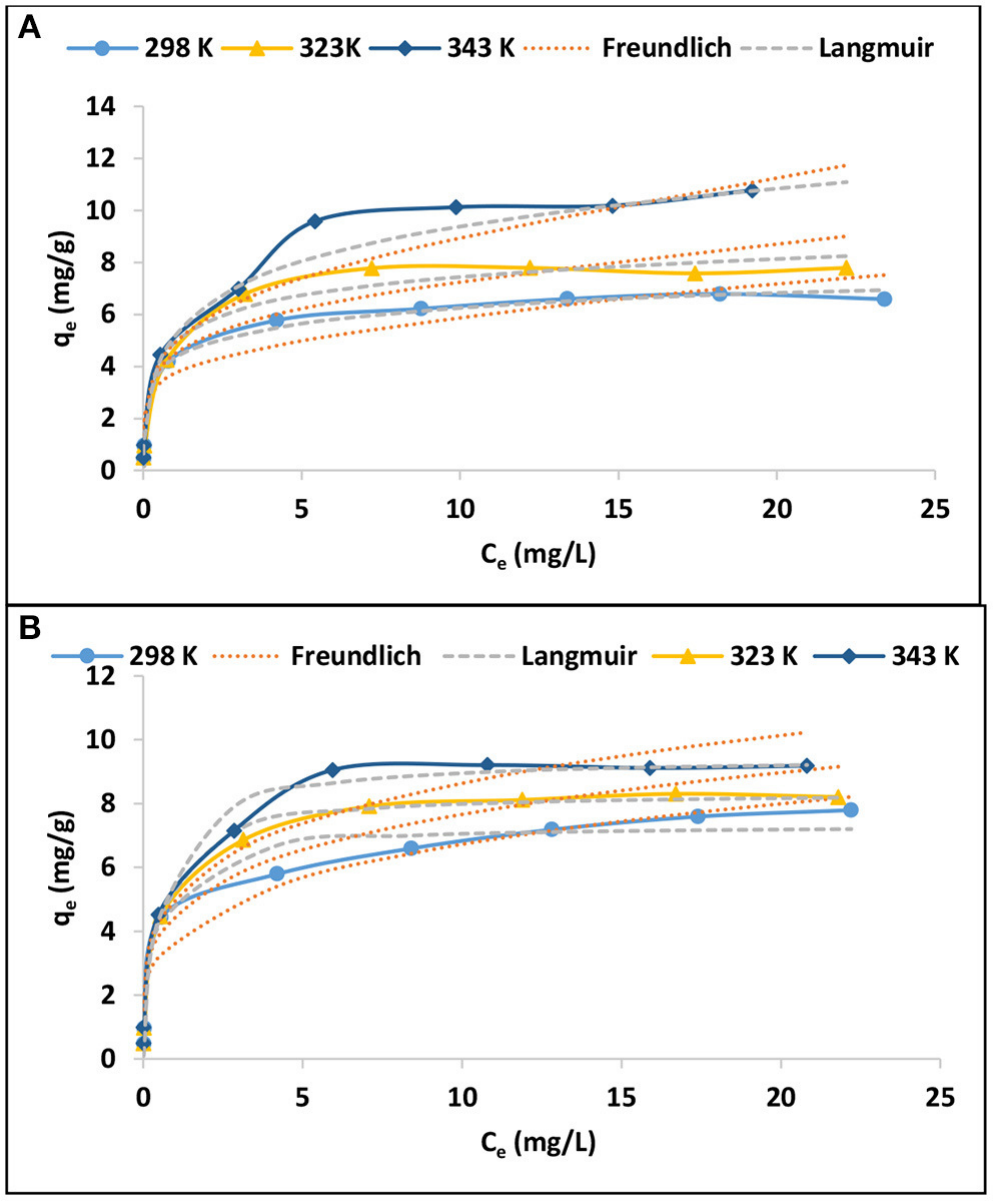
Adsorption isotherms for **(A)** As(III) and **(B)** As(V) (0.1 g/100 mL adsorbent dosage, 0.5–30 mg/L initial concentration, 60 min contact time, pH 6.5 ± 0.2).

The adsorption isotherms were evaluated by varying the initial adsorbate concentration from 0.5 to 30 mg/L. The experiment was conducted at 298, 323, and 343 K. The results are presented in Figures 6A,B in terms of equilibrium concentrations against adsorption capacity. As expected, the adsorption capacity increases with increasing equilibrium As(III) and As(V) concentration. Furthermore, the adsorption capacity increased with increasing temperature. Equations (4) and (5) of Langmuir and Freundlich adsorption isotherms, respectively, were used to explain the relationship between the adsorbent and the adsorbate (Tran et al., 2016).

(4) $$\begin{array}{l} q_{e}=\frac{q_{\max}bC_{e}}{1+bC_{e}} \end{array}$$

(5) $$\begin{array}{l} q_{e}=K_{f}C_{e}^{1/n} \end{array}$$

Where q_e_ (mg/g) is the adsorption capacity, C_e_ (mg/L) is the As(III) and As(V) concentration at equilibrium, b (L/mg) and q_max_ (mg/g) are Langmuir constants related to equilibrium adsorption constant and maximum monolayer adsorption capacity. K_f_ (mg/g) and 1/n are Freundlich constant values related to adsorption capacity and adsorption intensity, respectively. The nonlinear plots of Langmuir and Freundlich isotherms are presented in Figures 6A,B for As(III) and As(V), respectively while the models constant parameters are presented in Table 6.

**Table 6:** Langmuir and Freundlich adsorption isotherm parameters.

			**Langmuir**			**Freundlich**		
		**q_**e**__**exp**_ (mg/g)**	**q_**m**_ (mg/g)**	**b (L/mg)**	**R^**2**^**	**K_**f**_ (mg/g)**	**1/n**	**R^**2**^**
As(III)	298 K	6.79	7.99	0.21	0.98	1.88	0.4	0.90
	323 K	7.79	9.8	0.17	0.97	2.15	0.42	0.89
	343 K	10.77	12.15	0.09	0.99	1.96	0.52	0.95
As(V)	298 K	7.79	7.32	2.48	0.96	3.76	0.25	0.93
	323 K	8.2	8.36	2.10	0.99	4.52	0.22	0.93
	343 K	9.18	9.45	1.78	0.98	5.07	0.23	0.93

The adsorption isotherm data for As(III) and As(V) adsorption onto IOK was described by Langmuir isotherm model rather than Freundlich isotherm model. This suggest that the adsorption of As(III) and As(V) occurred on a mono-layered surface. The maximum theoretical adsorption capacities for As(III) were found to be 7.99, 9.88, and 12.15 mg/g at 298, 323, and 343 K, respectively while for As(V) adsorption capacities were found to be 7.32, 8.36, and 9.45 mg/g at these temperature ranges (Table 6). Conversely, the experimental adsorption capacities for As(III) at 298 and 323 K were found to be lower than those reported at the same temperature ranges for As(V).

#### Adsorption Thermodynamics

To further elucidate the adsorption mechanisms, thermodynamics parameters such as Gibbs energy change (ΔG°), the enthalpy change (ΔH°), and the entropy (ΔS°) were determined from Equations (6) and (7) (Singh et al., 2016).

(6) $$\begin{array}{l} \Delta G^{\circ }=-RT\ln\;K_{c} \end{array}$$

(7) $$\begin{array}{l} \ln\;K_{L}=-\frac{\Delta H^{\circ }}{\Delta RT^{\circ }}+\frac{S}{R} \end{array}$$

**Figure 7:**
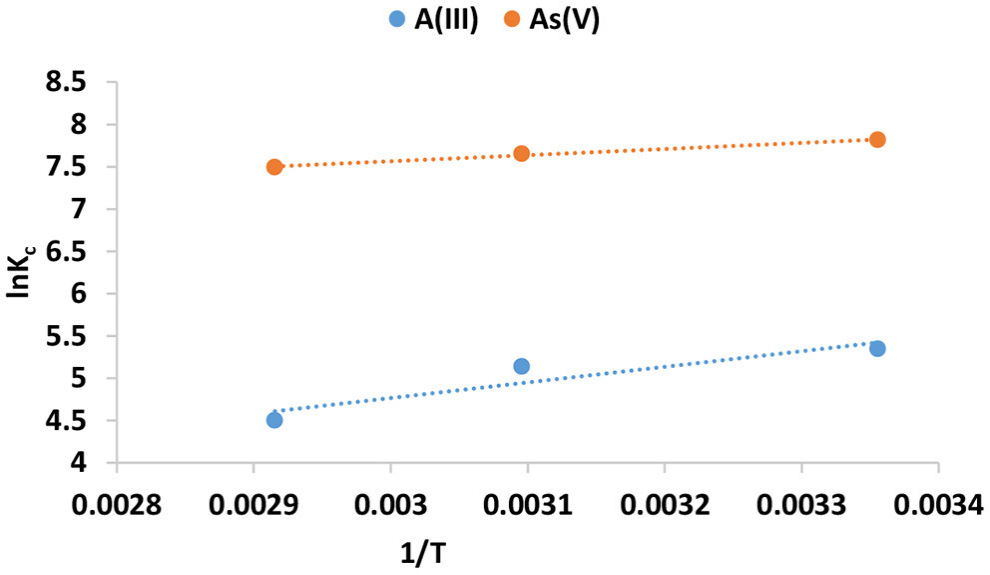
lnK_c_ as a function of reciprocal of adsorption temperature.

Where *R* is the molar gas constant, 8.314 J.mol^−1^K^−1^, T is the absolute temperature in Kelvin, ΔG° (KJ/mol) is the Gibbs free energy change. ΔH° (J/mol) is enthalpy change, ΔS° (J/mol) is the change in entropy and K_c_ is the dimensionless parameter derived from the Langmuir adsorption constant, b (L/mg) by multiplying b by the molecular weight of the adsorbate (Mw; g/mol), by 1,000 and then by 55.5 (number of moles of pure water per liter) (Tran et al., 2017). Values ΔH° and ΔS° of are determined from the slope and intercept of a plot of lnK_c_ against 1/T (Figure 7). The thermodynamic parameters are shown in Table 7.

**Table 7:** Thermodynamics parameters for As(III) and As(V) adsorption by IOK.

	**ΔG^**°**^ (KJ/mol)**	**ΔH^**°**^ (KJ/mol)**	**ΔS^**°**^ (J/mol)**
As(III)	298 K = −13.27 323 K = −13.79 343 K = −12.83	−15.35	6.4
As(V)	298 K = −19.36 323 K = −20.54 343 K = −21.35	−6.09	44.57

The value of enthalpy of change (ΔG°) for the adsorption of As(III) and As(V) onto Inorgano-organo modified kaolin clay mineral was found to be negative at both initial temperatures. This suggest that adsorption of As(III) and As(V) occurred spontaneously. The ΔH° value was found to be negative which indicating exothermic nature of the adsorption process. Exothermic reactions involves both physiosorption and chemisorption processes (Tran et al., 2016). The positive value of ΔS° suggest that As(III) and As(V) were randomly distributed on the surface of the adsorbent.

#### Effect of Co-existing Ions

**Figure 8:**
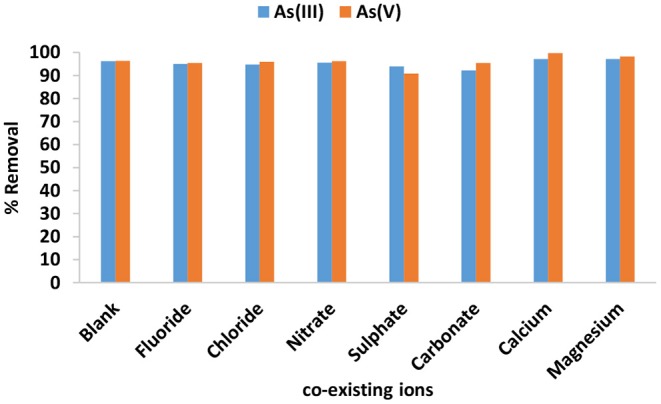
Effect of co-existing ions in the adsorption of As(III) and As(V) from the solution.

Figure 8 depicts the influence co-existing anions in adsorption of As(III) and As(V) by IOK. It is observed that the presence of Ca^2+^ and Mg^2+^ slightly increases the adsorption As(III) and As(V). This could be an indication that the presence of Ca^2+^ and Mg^2+^ makes the surface of the adsorbent to be more positively charged which consequently facilitate the attraction of As(III) and As(V) onto the created sorption sites (Qi et al., 2015). The presence of co-existing anions slightly inhabited the sorption As(III) and As(V). The adsorption of As(III) decreased significantly in the presence of carbonates while the adsorption of As(V) decreased significantly in the presence of sulfate. The decrease in percentage removal in the presence of anions could be attributed to competition for adsorption sites between the co-existing anions and arsenic species.

#### Regeneration Study

**Figure 9:**
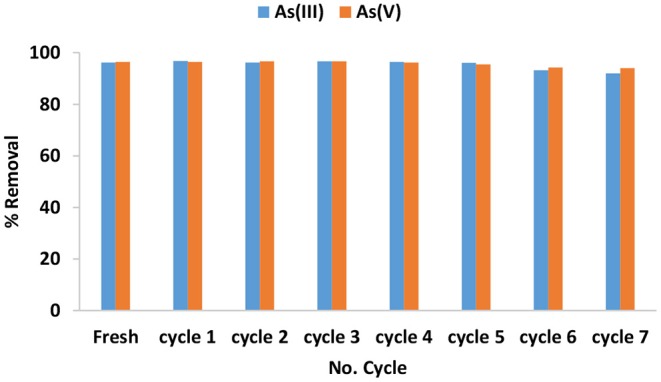
Variation of As(III) and As(V) removal as a function of regeneration-reuse cycles.

The regeneration and reuse of adsorbent was studied using 0.01 M HCl as a regenerating agent and the results for 7 successive cycles are presented in Figure 9. The percentage As(III) and As(V) removal achieved from the regeneration cycle 1 to cycle 5 was found to be >95% which is relatively equal to the percentage removal achieved from the virgin material. This could be an indication that treatment of the adsorbent with HCl increases the positive sites on the surface of the material. Slight decrease as the reuse-regeneration cycles continues to 7th cycle. The decrease could be due to inadequate regeneration of the sorption sites. This results suggests that IOK is a good material for use in arsenic removal from groundwater as it can be regenerated. The concentration of Fe and Mn were detected at trace concentrations below 0.1 mg/L in the filtrate after 7th cycle. Indicating that the adsorbent is stable.

#### Comparison With Other Adsorbents

Table 8 present the comparison for As(III) and As(V) adsorption capacities of different adsorbents reported in the literature with the maximum adsorption capacity obtained from the present study. From the table it can be noted that the maximum adsorption capacity obtained from the present study is quite higher as compared to those reported in the literature.

**Table 8:** Comparison of adsorption capacities.

**Adsorbent**	**Experimental conditions**	**q_**e**_ As(III) (mg/g)**	**q_**e**_ As(V) (mg/g)**	**References**
HDTMA-Al-bentonite	Initial concentration:2–18 mg/; Adsorbent dosage: 2 g/L; pH: 4.5	2.24	8.93	Lee et al., 2015
Iron impregnated charred GAP	Initial concentration: 0.05–200 mg/L; Adsorbent dosage: 0.5 g/100 mL; pH:7	3.25	5.09	Yin et al., 2017
Aluminum pillared HDTMA sericite	Initial concentration: 1 to 20 mg/L; adsorbent dosage: 0.2 g/100 mL; pH 4.5.	0.40	0.46	Tiwari and Lee, 2012
CTMAB-Fe-Montmorillonite	Initial concentration: 1–60 mg/L; adsorbent dosage: 0.1 g/25 mL; pH 6.5	11.36	8.85	Ren et al., 2014
Fe/Mn-HDTMA kaolin	Initial concentration: 0.5–30 mg/L; adsorbent dosage; 0.1 g/100 mL; pH:6.5 ± 0.5, Temp: 298 K	7.99	7.32	This study

## Conclusion

A clay based hybrid adsorbent for As(III) and As(V) was successfully synthesized through intercalation of Fe-Mn oxides and HDTMA-Br. Batch experiments showed that As(III) removal was optimum at pH range of 4-6 while the As(V) removal was optimum at pH range 4-8. The adsorption data for both species of arsenic fitted better to pseudo second order of reaction kinetics which suggest that the dominant adsorption mechanism was chemisorption. The isotherm studies showed that the data fitted better to Langmuir isotherm model as compared to Freundlich model indicating that adsorption of both As(III) and As(V) occurred on a monolayered surface. The maximum adsorption As(III) and As(V) capacity at room temperature as determined by Langmuir model were found to be 7.99 and 7.32 mg/g, respectively. The thermodynamic studies for sorption of As(III) and As(V) showed that values of ΔG° and ΔH° were negative indicating that adsorption process occurred spontaneously and is exothermic in nature. The regeneration study showed that the inorgano-organo modified kaolin clay mineral can be reused for up 7 adsorption-regeneration cycles using 0.01 M HCl as a regenerant. This findings showed that IOK developed in this study is suitable for use in removal of arsenic from groundwater.

## Data Availability Statement

All datasets generated for this study are included in the article/supplementary material.

## Author Contributions

RM designed and conducted the experiments, and also wrote the draft manuscript. MG made conceptual contribution, supervised the work, and edited the draft manuscript. PN made the conceptual contribution, made suggestions on the manuscript and supervised the work.

### Conflict of Interest

The authors declare that the research was conducted in the absence of any commercial or financial relationships that could be construed as a potential conflict of interest.
